# Exploring Pediatric Dermatology in Skin of Color: Focus on Dermoscopy

**DOI:** 10.3390/life14121604

**Published:** 2024-12-04

**Authors:** Emmanouil Karampinis, Olga Toli, Konstantina-Eirini Georgopoulou, Maria-Myrto Papadopoulou, Anna Vardiampasi, Efterpi Zafiriou, Elizabeth Lazaridou, Zoe Apalla, Aimilios Lallas, Biswanath Behera, Enzo Errichetti

**Affiliations:** 1 Second Dermatology Department, School of Health Sciences, Aristotle University of Thessaloniki, 54124 Thessaloniki, Greece; olgatolimail@gmail.com (O.T.); koneirgeo@gmail.com (K.-E.G.); myrto.papadopoulou4@gmail.com (M.-M.P.); bethlaz@auth.gr (E.L.); zoimd@yahoo.gr (Z.A.); 2 Department of Dermatology, Faculty of Medicine, School of Health Sciences, University General Hospital of Larissa, University of Thessaly, 41110 Larissa, Greece; zafevi@o365.uth.gr; 3 Department of Dermatology, Oncoderm Center One Day Clinic, 45332 Ioannina, Greece; 4 Department of Dermatology, General Hospital of West Attica “Agia Varvara”, 12351 Athens, Greece; 5 Department of Internal Medicine, General Hospital of Karditsa, 43131 Karditsa, Greece; 6 Department of Internal Medicine, General and Oncology Hospital of Kifissia “Agioi Anargyroi”, 14561 Athens, Greece; annavardiampasi@yahoo.gr; 7 First Dermatology Department, School of Health Sciences, Aristotle University of Thessaloniki, 54124 Thessaloniki, Greece; emlallas@gmail.com; 8 Department of Dermatology, and Venereology, All India Institute of Medical Sciences, Bhubaneswar 751019, Odisha, India; biswanathbehera61@gmail.com; 9 Institute of Dermatology, Department of Medical Area, University of Udine, 33100 Udine, Italy; enzoerri@yahoo.it

**Keywords:** pediatric dermatology, dermoscopy, skin of color, nevi, vascular lesions, molluscum contagium, scabies, warts, atopic dermatitis, psoriasis, tinea capitis, alopecia areata, juvenile xanthogranuloma, mastocytosis

## Abstract

This literature review aims to comprehensively evaluate the clinical and dermoscopic presentations of common pediatric diseases among children with skin of color (SoC) while also addressing potential variations based on racial backgrounds. This review encompasses various conditions, such as nevi subtypes, viral infections, infestations, and inflammatory dermatoses, as well as hair diseases and abnormal vascular formations, occurring in pediatric populations. Overall, we identified 7 studies on nevi subtypes, 24 studies on skin infections, 6 on inflammatory dermatoses, 10 on hair diseases and disorders, and 14 on miscellaneous disorders that also satisfied our SoC- and race-specific criteria. In case of no results, we assumed that dermoscopic findings are similar between SoC adults and children, confirming the hypothesis with our cases of dark-skinned Indian child patients. Inflammatory dermatoses such as psoriasis, eczema, and cutaneous mastocytosis, as well as skin infections like cutaneous leishmaniasis, appear with brownish backgrounds or exhibit dark structures more frequently than the respective dermoscopy images of Caucasian populations. Dermoscopy traits such as erythema in tinea capitis are uncommon or even absent on a dark-colored scalp, while a dark skin tone often obscures many characteristic features, such as dark and yellow dots in alopecia areata and even parts of an intradermal parasite in the case of scabies. Race-specific traits were also observed, such as corkscrew hair in tinea capitis, primarily seen in patients of African origin. Many dermoscopic images are consistent between SoC and non-SoC in various skin lesions, including vascular anomalies, juvenile xanthogranuloma, mastocytoma, and viral skin lesions like molluscum contagiosum, as well as in various hair disorders such as trichotillomania, while tinea capitis displays the most diverse reported dermoscopic features across SoC- and race-specific studies.

## 1. Introduction

Skin of color (SoC) refers to individuals with melanin-rich skin, encompassing various racial groups such as Hispanic/Latino, Asian, African, Native American, Pacific Islander, and multiracial backgrounds [1]. In dermatology research, there appears to be a relative neglect of SoC patients, as most studies have focused primarily on white-skinned populations. This phenomenon is also present in the field of dermoscopy, as the use of a dermoscope in countries in Africa has proven to be limited [2]. Other factors that broaden this gap are the deficiency of SoC dermoscopy images of skin lesions for educational purposes [3] and the common misunderstanding of the term SoC. SoC is often regarded as a group, while in fact it consists of significantly heterogenous populations in terms of skin color, with race-specific criteria playing an important role and outweighing the Fitzpatrick phototype system. However, limitations should also be acknowledged. Racial groups, particularly Hispanic and Asian populations, also include individuals with ‘light skin types’ (mainly phototype III). Therefore, analyses might be influenced by the presence of both fair and dark skin types (traditionally phototypes IV–VI) within these groups [4].

Black skin composition exhibits a larger keratinocyte size, greater keratinocyte density, reduced proteolytic activity, increased fibroblast numbers, and slower desquamation rates compared to that of Caucasians. Additionally, it has more cell layers in the stratum corneum compared to the thinner stratum corneum found in people of Asian descent. Considering these race-specific differences, it is expected that certain skin diseases would appear differently amongst different skin tones [5]. For example, in skin cancer, BCCs in Hispanic and Asian patients are more often pigmented, or at least display pigmented dermoscopic structures, such as maple-leaf-like and spoke-wheel areas, compared to those present in Caucasians [6,7,8]. Also, a rainbow pattern on dermoscopy has proved to be more frequent in dark-skinned individuals due to better contrast between the lesion and the surrounding skin [9].

The above-mentioned conclusions have led, over the last few years, to a growing interest in the use of dermoscopy in dark-skinned populations, as it may significantly increase diagnostic accuracy and reduce the need for biopsies. Reviews on skin of color focusing on the dermoscopy of cutaneous neoplasms [10], hair diseases [11], skin infections [12], and inflammatory dermatoses [13] revealed differences in the frequency of certain dermoscopy traits between Caucasian and SoC patients. Even in the developing field of artificial intelligence, AI programs give a different differential diagnosis of skin features when they are present on brown or dark-colored skin [14]. The need for dermoscopy knowledge in SoC is even more important in the pediatric population, in which performing invasive procedures may pose more difficulties.

Therefore, the purpose of this review is to provide a thorough overview of clinical and dermoscopic findings of the most common pediatric diseases in individuals with SoC while also assessing possible variations based on the patient’s racial background.

## 2. Materials and Methods

This review was carried out based on a comprehensive search of the literature which was performed through the PubMed electronic database from its inception to 1 July 2024. Titles, abstracts, and full texts were screened by two independent reviewers to select articles reporting dermoscopic features of skin disease in dark skin (Fitzpatrick’s phototypes IV–VI) in child populations or adolescent patients (patients under the age of 19 following the adolescence terminology of the WHO and the NICHD Pediatric Terminology [15,16]). Instances without histological diagnosis, non-English-language articles, reviews, personal opinions/editorials, and duplicates were ruled out. If information on the skin phototype or age of the patient was not provided, decision on inclusion was made based on the title/abstract/full text reporting that the manuscript concerned “dark skin” or “skin of color”.

Searches were carried out by 3 separate researchers and focused on the most common melanocytic and vascular growths, infections, and inflammatory, hair, and miscellaneous diseases encountered in the pediatric population according to practical reviews of dermoscopy in pediatric dermatology [17,18]. According to these reviews, the pediatric skin diseases presented in Table 1 were identified.

## 3. Results

### 3.1. Melanocytic Lesions

Regarding melanocytic lesions and SoC on dermoscopy in the child–adolescent population, we found seven studies: an Indian population-based, descriptive, observational study [19]; a Hispanic population-based cross-sectional survey with participants of multiple Fitzpatrick classes [20]; a prospective, cross-sectional study including individuals with skin types V and VI [21]; a descriptive, observational study on Spanish children [22]; a cross-sectional study on Brazilian children [23]; a case series with a retrospective qualitative analysis of dermoscopic features of common skin problems in Chinese children [24]; and a study on Colombian children grouped by the Akasu phototype categorization, mainly focusing on acral nevi [25].

#### 3.1.1. Acquired Melanocytic Nevi

Children with skin of color (SoC) typically exhibit fewer nevi in comparison to children with lighter skin tones. Among children with darker skin pigmentation, the prevailing dermoscopic pattern of nevi is a reticular pattern featuring central hyperpigmentation, while additional observable patterns include globular, homogeneous, and mixed patterns [19]. Based on cross-sectional data from a population-based cohort of Hispanic adolescents (mostly Fitzpatrick skin type IV) aged 11–13 years old, globular and reticular patterns were observed with almost the same frequency in Fitzpatrick skin type III, whereas the reticular pattern was the dominant dermoscopy image in Fitzpatrick types IV and V SoC children. The globular pattern was observed in all Fitzpatrick skin type II Hispanic children [20]. This high frequency of the reticular pattern was also noted by Lallas et al., 2011 [21], who examined nevi dermoscopy in a mixed-age SoC population, finding no statistical differences regarding age amongst the patterns, with a dark brown color being frequent in Fitzpatrick V individuals [18]. Also, the globular pattern is mostly linked with childhood and the reticular pattern with adolescence, a phenomenon also observed in Caucasian populations [21,22,23,24]

**Figure 1:**
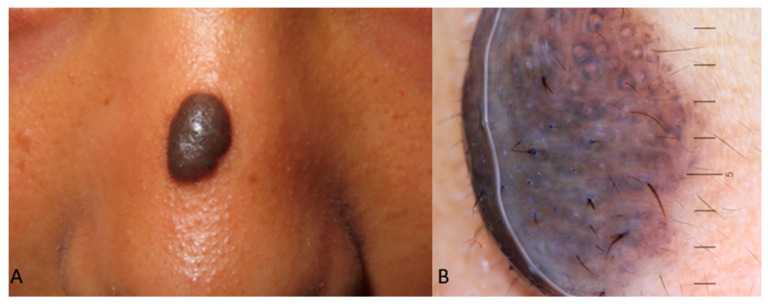
(**A**) Compound nevus on the nasal bridge of an Indian child presenting as a hyperpigmented nodule. (**B**) Dermoscopy of the lesion reveals perifollicular hypopigmentation and annular hyperpigmentation.

According to studies conducted in the Hispanic population, a uniform distribution of pigment was noticed in all nevus types [20,23]. Nevus size and nevus location also played an important role in the anticipated dermoscopy images, as a reticular pattern was mostly observed in the back region, as well as in small or medium-sized (>4 mm) nevi. The natural progression of nevogenesis from childhood to adolescence included an overall increase in the average number of nevi and dermoscopic changes such as a reduction in globules and pigmentation, as well as an increase in reticulation and pigmentation. Dermoscopy applied to nevi in children and adolescents of color may also reveal peripheral dots indicative of nevus augmentation, structureless areas, and, less frequently, branched streaks. Notably, nevi in different anatomic areas may present variations, with facial nevi presenting a pseudopigment network and dots [20]. A further division of acquired melanocytic nevi subtypes in a limited number of children with SoC aged 0–15 years old from an Indian population study was included in a study by Malladi et al., which presented junctional nevus dermoscopy with the same frequency of globular, homogenous, and mixed types, while compounds were reported with mixed and reticular patterns. The mixed subtypes in the junctional pattern were mostly globular and reticular, while in compounds, the respective combination was globular and homogenous. The pattern frequency changed with age [19] (Figure 1).

Acral acquired melanocytic nevi are a type of nevi that needs to be highlighted, as individuals with skin of color commonly exhibit a higher prevalence of nevi on their palms and soles [25]. Dermoscopy of acral nevi reveals three primary benign patterns in acral nevi: a parallel furrow pattern, characterized by pigment within the furrows, predominantly observed in individuals aged 0–12 years; a lattice-like pattern, featuring parallel brown lines connecting the pigment in the furrows; and a fibrillar pattern, characterized by streaks of pigmentation crossing the skin markings. Similar dermoscopy patterns were observed in Colombian children population expressed in Akasu categorization [25]. Additional dermoscopic patterns encompass the homogeneous pattern, frequently observed in SoC patients, presenting as a structureless brown area, the peas in a pod pattern, typically observed in acral congenital melanocytic nevi pattern and various non-typical patterns [25].

#### 3.1.2. Congenital Melanocytic Nevi (CMN)

The primary pattern observed in CMN is a cobblestone pattern, characterized by large, angulated globules resembling cobblestones. Malladi et al. presented findings from a case series involving Indian children, demonstrating that the predominant CMN pattern among their patients was the mixed–multicomponent type, encompassing globular–homogeneous and reticular–homogeneous patterns [19]. This was followed by homogeneous and globular–cobblestone patterns. Additionally, the observed features included milia-like cysts, target globules, target vessels, comedo-like lesions, hypertrichosis, skin furrow hypopigmentation, and perifollicular pigment changes. The presence of the halo phenomenon with leukotrichia may also be noted in CMN [19].

#### 3.1.3. Childhood Melanoma—Spitz/Reed Nevus

No data on the combination of childhood melanoma and SoC and dermoscopy were found. A case of Spitz nevi with a homogeneous pattern was found in the Indian population-based study of Malladi et al. [19].

### 3.2. Infectious Diseases

#### 3.2.1. Molluscum Contagium (MC)

**Figure 2:**
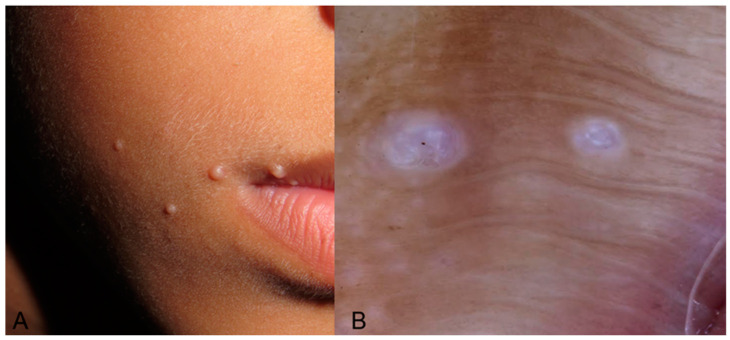
Molluscum Contagiosum presenting (**A**) as numerous, distinct, skin-colored pearly umbilicated papules on the face of a children. (**B**) Dermoscopy image revealing a purplish surface with shiny white amorphous structures or white clods.

Typically, MC lesions manifest as asymptomatic, skin-colored, firm, pearly umbilicated papules with a central core of keratin, frequently found on the face, trunk, extremities, and axilla, with sizes ranging from 2 to 5 mm. As per reports in children with SoC, MC may appear as dark, skin-colored, or purplish papules [26]. In the context of dermoscopy in SoC children, we found four case reports and one case series [27,28,29,30,31] primarily focused on lesions in the anogenital area; an examination of these lesions was in most cases conducted to rule out the possibility of sexual or child abuse. Dermoscopy of these lesions follows the classic characteristics observed in white individuals, including a smooth, reddish surface with shiny white amorphous structures or white clods. Most cases exhibited coronary and linear vessels along with reports of a crown vessel or red corona vessel distribution. However, the accurate characterization of vessels might be challenging due to the pressure induced by the dermatoscope on the lesion. All the case reports originate from Asian countries [27,28,29,30,31], primarily featuring fair or mixed phototypes, leading to dark skin being under-reported. A single case report documented an Indian boy with darker skin exhibiting multiple lesions on his face and neck. Notably, during dermoscopic evaluation, the examination revealed not only crown vessels and central yellowish structures but also the presence of rosettes in several lesions [32]. The dermoscopy of molluscum contagiosum in a general skin-of-color population [33,34], as reported by isolated accounts and images, highlights white globules as the main feature, often combined with peripheral dotted or linear vessels with branches. These observations are confirmed in our case presented in Figure 2.

#### 3.2.2. Scabies

Scabies, resulting from Sarcoptes scabiei mite infestation, has been recognized as a prevalent disease worldwide, and its diagnosis typically relies on clinical observation, with patients displaying a highly itchy papular or papulo-vesicular rash, accompanied by distinctive curvilinear burrows [35]. The clinical image of those lesions can be presented as raised dark skinned papules or whitish lesions in skin-of-color patients due to eczematization, with burrows being hard to distinguish on dark skin. In neonates, a more generalized papular or papulopustular eruption can be observed. Diagnosing scabies was traditionally based on optical microscopy by demonstrating the mite and its products externally on a glass slide. However, the systematic application of dermoscopy has focused on events occurring within the epidermis and the resulting morphological intricacies of Sarcoptes tunnels, including skin morphologies and unique dermoscopy characteristics. The specific dermoscopy features of scabies detected in individuals with skin of color can also aid in diagnosis.

**Figure 3:**
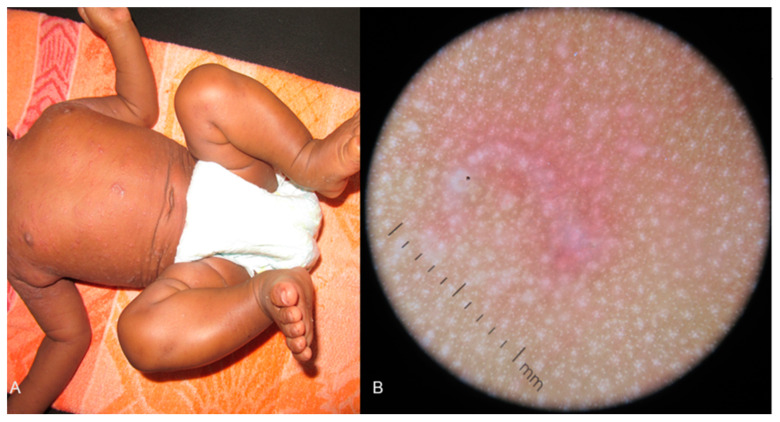
(**A**) Diffuse papular or papulovesicular rash on the trunk and extremities of an infant diagnosed with scabies. (**B**) The dermoscopy image presents a silver-gray translucent tunnel, known as a “jet with contrail sign”, and a black-colored triangle structure at the end of the tunnel called the “hang glider” sign or “triangle” sign.

Six studies (two case series and four case reports) [36,37,38,39,40,41] reporting findings on scabies in pediatric populations were detected. Five out of the six studies addressed fair-skinned Asian populations; therefore, the unique characteristics of dark skin cannot be assessed adequately. The main findings of scabies observed include a silver-gray translucent tunnel, known as a “jet with contrail sign”, and a dark brown- or black-colored triangle structure at the end of the tunnel called the “hang glider” sign or “triangle” sign (Figure 3), which correlates to the gnathosoma and the two anterior pairs of the forelimbs of the scabies mite [32,33,34,35,36,37]. Additionally, another specific characteristic of scabies—the “wake sign”, which corresponds to an inflamed area behind the mite with abnormal keratinization and points towards the position of the mite—was detected in one case report [40]. It is noteworthy to highlight that in dark-skinned patients with scabies, nonspecific pigmented areas without distinct structures are frequently observed around the burrows due to the evolution or settling of a post-inflammatory hyperpigmentation [41]. Worth mentioning is that the diffuse scaling resulting from intense scratching or the dominant pigmented background in skin of color may render the detection of the mite or tunnel parts on dermoscopy and of classic dermoscopy signs challenging. For example, the pigmented part of a burrow is often missing on dermoscopy [41].

#### 3.2.3. Viral Warts

Skin of color and race play a significant role in the occurrence of warts, with a higher reported frequency in white children, while non-Caucasian races have been associated with a faster resolution of the lesions [42]. Children with skin of color, as indicated by a clinical epidemiology study, tend to appear with mostly plane-type and plantopalmar warts when under the age of 10, while 11–20-year-old Indian children mainly appear with the plantopalmar, plane, common, and mosaic wart subtypes. The location of the warts detected was the upper limbs [43].

**Figure 4:**
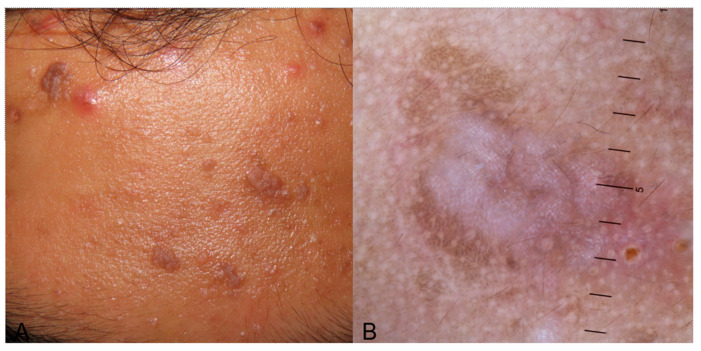
(**A**) Plane warts in a young dark-skinned boy presenting as multiple well-demarcated brownish papules or plaques. (**B**) Dermoscopy image exhibiting white-gray globules and clods on a brown background.

Concerning the dermoscopy traits of verruca vulgaris, there has been no primary research (case report, case series, case–control studies, etc.) including exclusively dark-skinned pediatric populations, and there is only one case series including seven Chinese children with warts [24]. Their dermoscopy features included thrombosed capillaries presenting as black-to-red dots, a papilliform surface, and interrupted skin lines. Information can also be extracted from studies on the dermoscopic features of warts in darker skin types IV–V, irrespective of age. Agarwal et al. described that in verruca vulgaris, the most common background color was brown, followed by pink and yellow. The majority of common warts are well defined with skin markings, while the presence of hemorrhage and crusting is not common. Brown dots, dotted vessels, and black dots/globules indicative of thrombosed vessels are common dermoscopy features of common warts [44]. Papillary projections and papilla structures, as well as perivascular white halos, can be additional clues in the dermoscopy of common warts, creating a ‘frogspawn’ pattern, which can also be observed in SoC [45]. On the contrary, palmoplantar warts can be characterized by irregularly distributed hemorrhagic reddish-brown dots or globules due to high pressure at the sites of occurrence [46]. Concerning background color, a brownish background is a predominant characteristic in plane warts (Figure 4), while a yellow background is mainly observed on palmoplantar lesions. Plane warts are the least defined wart lesions, as indicated by the macroscopic image in Figure 4.

#### 3.2.4. Tinea Capitis (TC)

TC is a superficial fungal infection caused by dermatophytes that parasitize the scalp and the hairs on the head. It is particularly common among children in West Africa and parts of Asia, including India. This high frequency is attributed to a humid climate, poor hygiene, poor living standards, and certain social habits; therefore, dermoscopy is a method that warrants a rapid examination technique. SoC children tend to have naturally curly hair with different hair densities [47] and are therefore expected to have distinct characteristics concerning trichoscopy images of scalp diseases.

We found seven African, three Indian, and one Asian race-specific studies including TC dermoscopic features in pediatric populations. A study from Nigeria included non-inflammatory and inflammatory clinical types of TC, ranging from a gray patch TC subtype and the black dot to the favus and kerion, as well as including co-existing subtypes. Perifollicular scaling and interfollicular scaling were the commonest findings, followed by black dots. Regrowing hairs, comma hairs, and broken hairs were less frequently seen, while corkscrew hairs, vellus hairs, zigzag hairs, and peripilar casts were present in even lowest percentages [48].

Contrary to the above-mentioned study, comma and corkscrew hair types were predominant in two case reports [49,50] compared to scaling, while in a small case series (including one Ethiopian, one Egyptian, and one Congolese child), corkscrew hair was not observed [51]. Another study that confirms the predominance of comma hair is a case series from North Africa [52]. According to a larger study by Brasilero et al. [53], the most common dermoscopic feature in tinea capitis with positive culture was scaling (diffuse or perifollicular) and broken hair, and there was an association between positive tinea capitis culture and the presence of scaling. In this study, corkscrew hair was more frequent compared to comma hair. A further differentiation of tinea capitis in African children was performed, associating endothrix and exothrix infections with specific dermoscopy traits. Specifically, comma hair and corkscrew hair were associated with endothrix infections, while barcode-like hair and zigzag hair with exothrix and the combination of ectothrix and endothrix infections with mixed hair features [54].

**Figure 5:**
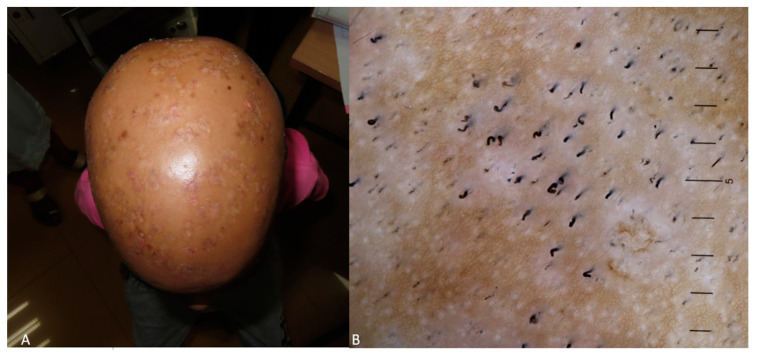
(**A**) Tinea capitis in a dark-skinned child presenting as red-to-dark-brown patches spread across the scalp, resulting in complete scalp hair loss. (**B**) Dermoscopy image exhibiting black dots, comma hairs, corkscrew hairs, and a few vellus hairs. The findings in this case are consistent with those reported in studies.

Concerning patients of Indian origin, comma hairs were also the predominant dermoscopy characteristic (12 patients), followed by corkscrew hairs, zigzag hairs, black dots, short vellus, barcode (morse code hairs), and scaling [55]. Comma hairs and black dots were the most common in Indians, and comma hair detection was useful in differentiating between alopecia areata cases in the pediatric population, as no comma hairs were found in those cases [56]. Comma hairs, followed by black dots and coiled hair, were also the dominant tinea capitis dermoscopy feature in the Indian pediatric population in another study [57] (Figure 5).

Regarding light-skinned Asians, the most sensitive findings in TC were broken hairs, interfollicular and perifollicular scales (100.0%), black dots (77.8%), and diffuse erythema (44.4%) [58]. It is worth mentioning that the presence of abundant melanin makes erythema hard to observe in SoC patients. Concerning Hispanic patients, a case of tina capitis was also observed in a Hispanic woman, with similar dermoscopy images expected in Hispanic pediatric populations. Apart from comma hairs, the patient—although straight-haired—presented with corkscrew hairs, indicating that this trait can be observed not only in African or curly-haired individuals [59]. However, more studies are needed to evaluate the dermoscopy findings of tinea capitis in Hispanic patients. Similar trichoscopy patterns have been identified in mixed-race populations from the Dominican Republic and Mexico, with comma and corkscrew hairs and black dots being the main findings [60].

#### 3.2.5. Pediculosis Capitis

Regarding the dermoscopy of skin of color in cases of pediculosis, we identified only two narrative reviews that relied on dermoscopy images of scalp pediculosis in the Indian population [45,61]. In these instances, dermoscopy shows, in most cases, brown-colored live nits and/or empty or abortive nits attached to the hair shaft with a translucent and crystalline whitish structure, respectively, or/and, less frequently, live head lice with blood meals. It is important to recognize the difficulty of identifying brown-colored structures against a brown scalp background and distinguishing nits from ‘pseudonits’, which are often dandruff flakes. Unlike nits, these flakes are irregularly shaped and not adherent to the hair shafts [43,59].

#### 3.2.6. Cutaneous Leishmaniasis

Cutaneous leishmaniasis is endemic in Central and South America, Africa, Asia, and Southern Europe. Acute lesions of cutaneous leishmaniasis may present as papules, nodules, or plaques which are crusted or ulcerated, making the disease a great imitator. A clinical and dermoscopy association has been established. Yellow tears and structures, as well as perilesional hypopigmented halos, are more common with papule forms, white starbursts are more frequent in ulcer subtypes, and salmon-colored ovoids are a dermoscopy feature of granulation reactions in all forms [62]. Of interest is the variability in vascular patterns that cutaneous leishmaniasis can present dermoscopically, as irregular linear, tree-like, hairpin, glomerular, and/or comma vessels have been observed in leishmaniasis’s various clinical forms [62].

Studies that include SoC pediatric populations come from Indian and Hispanic populations, while large-scale studies across all age groups are mainly conducted in Turkish populations, as the disease is endemic in a large proportion of country [62]. Follicular plugs, white lines, and ulcerations were indicated as the main dermoscopy traits in SoC patients in a case–control study by Errichetti et al. including 14 SoC cases [33].

Concerning SoC children, in an Indian case series, a girl presented with a nodular lesion on her forearm, with the dermoscopy image showing a white starburst pattern, a central red area on a pinkish-white background, and comma-shaped and linear vessels. Another case in the same study involved a boy with an erythematous plaque, where dermoscopy revealed a starburst pattern of radially arranged chrysalis strands and linear vessels [63]. In Brazilian populations, leishmaniasis dermoscopy included general erythema, ulceration features (microulcerations and central ulcers), keratin and collagen structures (focal structureless white areas, hyperkeratosis, follicular plugs), and perilesional white halos and polymorphic vessels [64]. Such dermoscopy traits are in line with the dermoscopy presentation of an erythematous plaque in a seven-year-old Spanish boy [65]. Based on those cases of pediatric patients, 30, 59, hyperkeratotic structures, erythema, and polymorphic vessels seem to be the predominant dermoscopy traits of cutaneous leishmaniasis.

### 3.3. Inflammatory Diseases

#### 3.3.1. Atopic Dermatitis (AD) and Eczema

**Figure 6:**
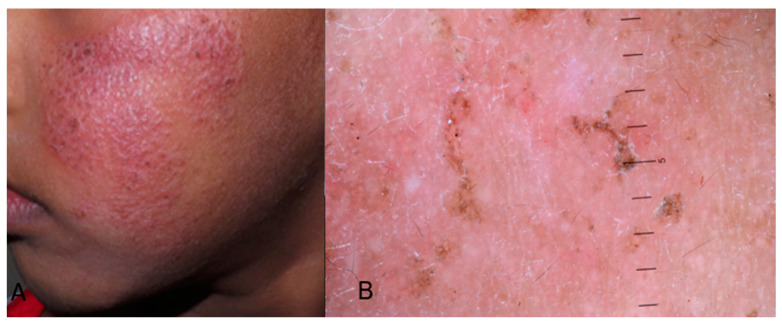
(**A**) Atopic dermatitis presenting as an erythematous, violaceous, well-demarcated plaque on the left cheek of a child with skin of color. The lesion manifests with prominent scales and lichenification. (**B**) Dermoscopy image revealing white and brown scales, dotted vessels with a patchy pattern, brown black dots, clods and lines, a brownish-pinkish background, and adherent fabric fibers.

Atopic dermatitis (AD) in individuals with skin of color (SoC) adheres to the typical progression pattern of the disease. Infantile AD, occurring before the age of 2, typically manifests as acute and/or subacute eczematous dermatitis on the cheeks (Figure 6), neck, scalp, and extensor surfaces, while childhood AD is characterized by subacute and chronic lichenified skin lesions and primarily involves the neck, flexural areas, wrists, and ankles. The only data regarding dermoscopy of dermatitis and eczema in patients with dark phototypes come from adult or mixed-age populations [13]; one case series study on Chinese children was reported [24].

Assuming uniformity in the clinical and dermoscopy traits of core lesions between SoC children and adults, a parallel approach can be adopted when evaluating individuals with skin of color, acknowledging the distinction that adults often present with more lichenified lesions due to chronic eczema [66]. Lesions in SoC children can be violaceous, ashen gray, and darker brown. The difference with the respective dermoscopy images of dermatitis in white people is based on the presence of brown pigmented structures (such as a reticular pattern, lines, globules, and clods) indicative of progressing pigmentary incontinence combined with classic atopic dermatitis dermoscopy features (scales and dotted vessels) [33,67] (Figure 6).

The most characteristic dermoscopic finding are patchy yellow, dirty brown, or white scales and crusts. Other findings concern purple, brown, or black dots, dotted vessels, or red globules with a patchy pattern and adherent fabric fibers. The background is usually brownish black or red/pinkish, and erosions may coexist [63]. These characteristics are confirmed by our case presented in Figure 6. Race-specific variations may influence dermoscopy images. Asian individuals often exhibit lesions with well-defined borders, resembling psoriasis plaques, and show increased scaling and lichenification compared to their white counterparts with AD [66].

#### 3.3.2. Psoriasis

**Figure 7:**
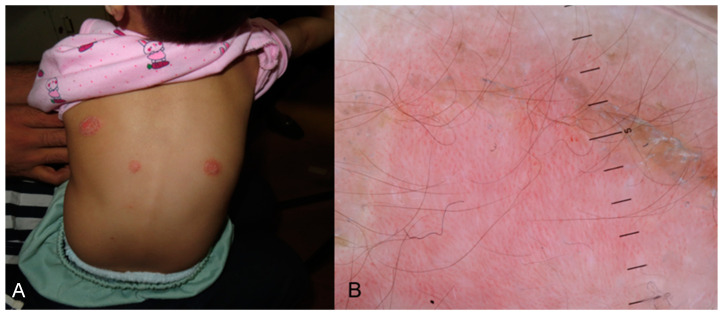
(**A**) Erythematous, ovoid, well-demarcated plaques on the back of a child with skin of color indicative of plaque psoriasis. (**B**) Dermoscopy image exhibiting white scales, brown blotches and structureless areas, and red globules/dotted vessels with a regular distribution on a dull red/pinkish background. A specific feature in this image is the “red globular ring pattern” which concerns vessels, specifically red globules with a network-like arrangement.

Due to an inadequacy of studies on dermoscopy in pediatric psoriasis in skin of color, information comes from studies performed in adults with skin of color, focusing more on the locations and clinical variants found in children. Psoriasis is a disease with a significant prevalence of vascular structures attributed to considerable epidermal acanthosis, which makes the background of the skin lighter; this is also found in pediatric psoriasis [68]. The most frequent dermoscopic finding is dotted vessels in a regular and symmetrical pattern and white scales with a patchy or diffuse distribution. Other findings are a light or dull red background and pigmented structures (brown, gray, or blue structureless areas, dots, globules, blotches, or clods) [33,69]. The same dermoscopic features can also be detected in pediatric patients (Figure 7). In an Asian study in children, those findings were also confirmed [24]. A globular ring pattern with vessels distributed in a network-like arrangement and a dermoscopic Auspitz sign can also be occasionally observed (Figure 7).

#### 3.3.3. Pityriasis Rosea (PR)

PR is an acute, self-limiting disease characterized by erythematous scaly papules and plaques. A yellow-orange background, dotted vessels, a patchy vessel distribution, and yellow white scales are the most frequent dermoscopy features in both Caucasian and Indian patient populations, while a violaceous background with white scales, pigmentary changes, and punched-out pits has been noted in patients of African origin [70,71]. The only race-specific study that involved dermoscopy in a SoC pediatric population was a cross-sectional study of the epidemiological and clinical aspects of PR in the Indian population [70]. The study involved mainly PR dermoscopy images including a diffuse red-to-yellow background with a peripheral collarette and peripheral dotted vessels, aligning with the predominant dermoscopy features found in the general Indian population. More studies are necessary to confirm that the race-specific characteristics and dermoscopy of lesions in adults and children are similar in cases of PR.

#### 3.3.4. Mastocytosis

Mastocytosis is a disorder characterized by the accumulation of mast cells in various organs, most commonly in the skin. Urticaria pigmentosa is the most common form of mastocytosis and it often develops in infancy or early childhood. We found two studies that included dermoscopy in SoC pediatric dermatology—one case series with two Fitzpatrick type IV instances from India [72] and one Spanish retrospective study that included age-specific dermoscopic characteristics of urticaria pigmentosa [73]. Concerning the first study, a 7-year-old boy presented with hyperpigmented macules over the back with positive Darier’s sign. Dermoscopy revealed dark brown lines in a dark and thick reticulate pattern, which is an exaggeration of the pigment network seen on normal skin. The second case was a girl with multiple brownish skin lesions, predominantly over the trunk. Dermoscopy in that case showed brownish reticular thick lines in the form of pigment network [72]. According to a large-scale study with 61 children, light brown and yellow-orange blots were more frequent than the pigment network found in the previously mentioned Indian study. However, the yellow-orange pattern was more associated with solitary mastocytoma rather than the maculopapular form, indicating the importance of agreement between clinical and dermoscopy findings. Also, another pattern—reticular–vascular—was also observed, but it was less frequent in children compared to adults [73].

### 3.4. Hair Diseases

#### 3.4.1. Childhood Alopecia Areata

**Figure 8:**
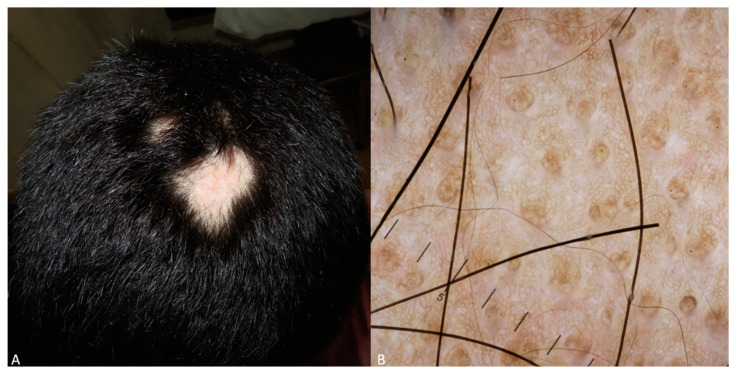
(**A**) Alopecia areata in a dark-skinned child, presenting as well-defined patches of hair loss on the scalp. The area is smooth and lacks visible scaling or inflammation, in sharp contrast to the surrounding hair, which appears healthy and unaffected. (**B**) Dermoscopy image exhibiting numerous yellow dots, as well as black dots and vellus hairs, on a brown-colored background.

Alopecia areata is a type of autoimmune non-cicatricial alopecia. Race-specific data concerning childhood alopecia areata were found in three retrospective cross-sectional studies, two on the Indian population and one multi-age study on the Asian population. Yellow dots were found to be the commonest finding, present in 88% of cases, followed by short vellus hairs and black dots in 76% and 28% of children, respectively, while exclamation marks were the rarest characteristic [74] (Figure 8). On the contrary, according to the second Indian population study, black dots and exclamation mark hairs were found at a higher frequency, along with off-white dots. Yellow dots are generally less frequent in children because the sebaceous glands are not completely developed before puberty. This fact is confirmed by a study by Bapu et al. [75], which states that yellow dots are more prominent in SoC patients aged over 20 years old. On the other hand, black dots are indicative of disease activity, and they can be age-independent. However, this disease activity correlation is not proved by certain studies [75]. Yellow and black dots, as well as exclamation, vellus, and broken hairs, were found on trichoscopy in a mixed-age study in the Asian population [76].

#### 3.4.2. Trichotillomania

According to the previously mentioned mixed-age Asian study concerning alopecia areata, dermoscopic features in cases of trichotillomania frequently include broken hairs of different lengths and black dots. Yellow dots were found to be a significant indicator distinguishing alopecia areata from trichotillomania [56]. Broken hair and black dots also proved to be the most common findings in an Indian pediatric population study [56]. 

### 3.5. Miscellaneous

#### 3.5.1. Juvenile Xanthogranuloma (JXG)

JXG is the most common type of non-Langerhans cell histiocytosis; it is marked by yellow, red, or brown papulonodular lesions that typically appear in infancy and childhood. The main dermoscopy findings include mainly orange-yellow background coloration accompanied by a setting-sun appearance, clouds of paler yellow globules, whitish streaks, and branched and linear vessels [75]. Dermoscopy images vary according to the evolution of the lesion, a phenomenon that is present regardless of skin color, with the setting-sun appearance absent in late regressive stages. We found race-specific and SoC studies (one Indian case series, one case report on Hispanic patients, two large-scale Asian descriptive cross-sectional studies, and one Asian case report [77,78,79,80,81]). Concerning Asian populations, a descriptive study which included 41 pediatric patients and a case series-based review including five pediatric cases both concluded that the “setting sun” pattern holds diagnostic value in the early and classic stages, while the “clouds” of paler yellow areas are more common in the classic and transitional stages [77,81]. The setting-sun pattern, which is recognized as an orange-yellow background with a subtle peripheral erythematous border with linear branched vessels, was also observed in small case reports in Korean [78] and Spanish children [79], and it is a typical dermoscopy trait irrespective of ethnicity. The same color pattern is present in the only case series on dark-skinned patients, which is based on Indian patients, with four of them under 18 years old. In this case, JXGs appeared as brown plaques or nodules that on dermoscopy appeared with a yellowish-orange center with perifollicular pigment.

#### 3.5.2. Vascular Anomalies

##### Infantile Hemangiomas (IHs)

Despite the fact that IHs represent the most prevalent tumors during infancy, the only dermoscopic information accessible originates from two case series conducted in Asia [24,82] and a case report detailing an IH in a Sudanese infant [83]. On dermoscopic examination, IH may manifest as red lacunae, accompanied by or without septa, against a background of red or red–blue color. Additionally, distinctive vascular structures, such as serpiginous, coiled, or linear–curved vessels, may also be present alongside lacunae.

##### Port Wine Stains (PWSs)

**Figure 9:**
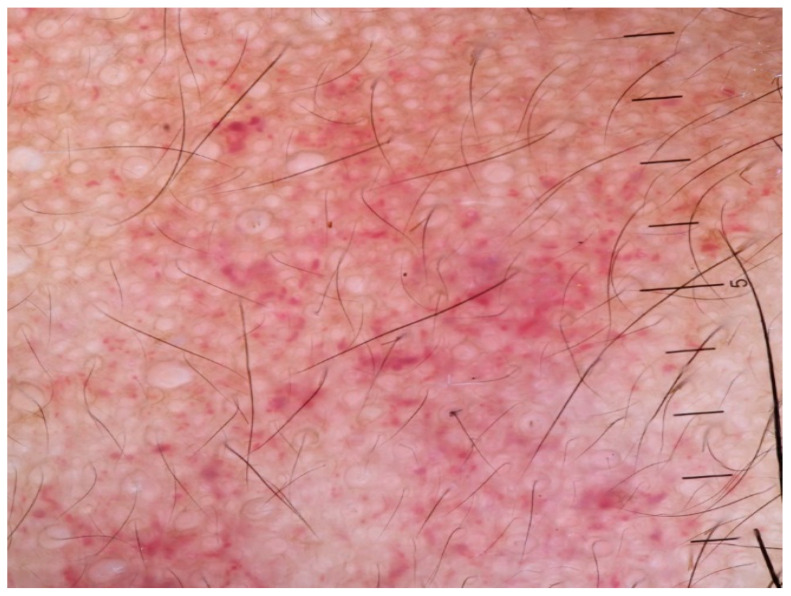
Dermoscopy image of a PW lesion in a skin-of-color child revealing a mixed vascular pattern with globular and reticular types of vessels.

Two Asian case series provide insights into the primary dermoscopic patterns associated with Port Wine Stains (PWSs) in pediatric patients with SoC [24,84]. Dermoscopic examination of PWSs may uncover either a globular or a reticular vascular pattern, with the most prevalent pattern being a mixed one, consisting of both globular and reticular components. Research by Huang et al. [84] indicates that the predominant subtype in children is the pink type, whereas purple and thickened types are more prevalent in adults. Additionally, facial lesions may exhibit the presence of white circles (Figure 9).

##### Pyogenic Granuloma (PG)

No research exclusively focuses on the dermoscopic features of PG in pediatric cases. However, three studies encompassing both children and adults with SoC note that dermoscopy clues of pyogenic granuloma in skin of color are as follows: a red or red-whitish homogeneous area featuring a white collarette, white rail lines, and vascular structures. Additionally, dermoscopic observations may include white scales, serum crusts, and blood spots [82,85,86].

## 4. Discussion

Dermoscopy in SoC reveals differences and similarities in dermatoses concerning pediatric populations. Backgrounds in inflammatory dermatoses such as psoriasis, eczema, and mastocytosis [72] or skin infections such as cutaneous leishmaniasis [63] appear brownish or feature dark or brown structures more often than in Caucasian populations. Clues such as erythema in tinea capitis are not prevalent or even absent on a dark-colored scalp. Indeed, dark skin makes many characteristic features, such as dark and yellow dots, nail discoloration, and even parts of an intradermal parasite, harder to distinguish. Also, nevi subtypes appear darker or dark brown, with reticular being the main pattern encountered [20,23]. Race-specific characteristics were also noted, such as the presence of corkscrew hairs in tina areata, mainly in patients of African origin.

The same dermoscopy features in SoC and non-SoC appear in many skin lesions, including vascular anomalies, JXG, mastocytoma, viral skin lesions such as MC and morphea, etc. Also, hair diseases such as alopecia areata and trichotillomania appear with the same disease-specific dermoscopy signs, with tinea capitis appearing with the most scattered dermoscopy features reported in SoC and race-specific studies [48,49,50,52,53,54].

The presence of disease mimickers plays an important role in pediatric dermatology, making the significance of dermoscopy even greater. Due to extensive playtime and activities that are strenuous to the feet, pediatric populations (both white and SoC) may develop calluses or even corns; therefore, their differential diagnosis against palmoplantar warts is important. Unlike palmoplantar warts, a plantar corn or callus is characterized by a translucent and uniformly opaque central core without the presence of hemorrhagic structures and with retained dermatoglyphics. However, in individuals with SoC, pseudo-hemorrhagic effects may be present and can be misleading due to the attachment of dirt particles to the skin, compounded by the pigmented nature of the skin lesion [45].

Another example of a potentially confusing mimicker can be the case of atopic dermatitis in SoC children. In children of African descent, atopic dermatitis may present as papular lesions with a lichen planus-type presentation; therefore dermoscopy can help in the differential diagnosis [66,87]. Additionally, dark-skinned children may manifest hyper- or hypopigmentation disorders, resulting in dyschromia due to post-inflammatory reactions. Worth mentioning is the high prevalence of terra-firma forme dermatosis (dirty dermatitis) in SoC children with atopic dermatitis; therefore, the differentiation of those dermatitis cases can be based on clearing with an isopropyl or 70% ethyl alcohol swab or by dermoscopic observation of large polygonal plate-like brown scales arranged together, producing a mosaic or cobblestone pattern [88,89].

The following table (Table 2) summarizes comments comparing the similarities and differences between pediatric patients with SoC and with light skin.

## 5. Conclusions

This study has identified both differences and similarities in how dermatoses among pediatric populations present on dermoscopy in skin of color (SoC). Additionally, the diverse dermoscopic features observed across different races and SoC children should be recognized and taken into account when examining skin diseases in pediatric patients.

## Figures and Tables

**Table 1:** The most common skin entities included in pediatric dermatology.

Melanocytic Lesions	Skin Infections/Infestations	Inflammatory Skin Disorders	Hair Disorders	Miscellaneous Disorders
Acquired Melanocytic Nevi Spitz/Reed Nevus Congenital Nevi Childhood Melanoma	Molluscum Contagiosum Warts Tinea Capitis Scabies Pediculosis Capitis Cutaneous Leishmaniasis	Eczema Psoriasis Pityriasis Rosea Mastocytosis	Alopecia Areata Trichotillomania	Juvenile Xanthogranuloma Vascular Anomalies Pyogenic Granuloma

The search on each category included “dermoscopy” OR “dermatoscopy” OR “pigmentaroscopy” AND “children” OR “pediatric” OR “infants” OR “adolescence” AND “skin of color” OR “dark skin” OR “black skin” OR “ethnic skin” OR “dark phototype” OR “African skin” OR “Indian skin” AND “DISEASE”. The disease was each time one of the diseases presented in each column.

**Table 2:** Observations derived from the comparison of the similarities and differences between pediatric patients with SoC and with light skin.

Most Common Skin Entities in Pediatric Dermatology	Comparison Between SoC and Light Skin in the Most Common Skin Entities Encountered in Pediatric Dermatology
Melanocytic Lesions	
Acquired Melanocytic Nevi	In skin of color, nevi may exhibit deeper pigmentation and frequently display reticular patterns, particularly in individuals with darker Fitzpatrick skin types. Both populations experience similar age-based nevi pattern shifts. Uniform pigmentation in SoC is typical, contrary to light skin, in which nevi may present with atypical characteristics
Congenital Melanocytic Nevi (CMN)	On dermoscopy, CMN in both groups frequently display hypertrichosis, perifollicular pigmentation, and cobblestone-like globules. In SoC dermoscopy, CMN may exhibit darker pigment variations and more noticeable perifollicular pigmentation, Lighter skin tones often show more distinct pattern contrasts.
Infectious diseases	
Molluscum Contagium (MC)	MC in both populations shows rosettes (under polarized light) and crown vessels on dermoscopy. SoC dermoscopy images in MC often includes additional yellowish central structures.
Scabies	In dermoscopy images of scabies on SoC, the normal pigmentation of children’s skin can obscure the visibility of burrows and tunnels. Dermoscopy features of scabies such as inflammation erythema and classic signs such as the Kite sign and Delta wing signs are more common on fairer skin.
Viral Warts	Black dots are less distinct in SoC due to the darker background color. Both populations share the “frogspawn” and papilliform dermoscopy patterns. Palmoplantar variations in SoC often exhibit a more yellowish hue under dermoscopy.
Tinea Capitis (TC)	TC in SoC frequently presents with corkscrew and comma hairs. Black dots are a consistent finding in both groups but can be masked by scaling or erythema. Different frequencies of trichoscopy findings were noted among patients with skin of color, with African populations showing a higher prevalence of corkscrew hair features.
Pediculosis Capitis	In SoC, live nits may be harder to detect against a darker scalp. In both groups, live versus empty nits can be distinguished based on color and shape.
Cutaneous Leishmaniasis (CL)	SoC dermoscopy images in CL display similar patterns, though the hyperpigmentation and hyperkeratosis encountered in SoC patients may complicate visualization of classic starburst signs. Polymorphic vessels and erythema in CL are common dermoscopy features in both groups.
Inflammatory Diseases	
Atopic Dermatitis (AD) and Eczema	SoC dermoscopy images in AD present with brown scales and a more complex background color, while dotted vessels and serocrusts are characteristic traits in both groups. Differences in scaling and pigmentation between race groups can affect lesion appearance and distribution.
Psoriasis	Dermoscopy of psoriasis may show additional pigmented structures (brown, gray, blue) not typically seen in lighter skin, complicating visual diagnosis. White scales and dotted vessels remain common dermoscopy findings across skin types.
Pityriasis Rosea (PR)	Dermoscopy of PR in both SoC and lighter-skinned patients reveals peripheral collarettes and dotted vessels, with a red-to-yellow gradient more frequently encountered in SoC individuals.
Mastocytosis	In both populations, mastocytosis may display a reticular network under dermoscopy. In case of SoC, the network observed in Indian children may present with a more intense pigment, while Hispanic patients may have a more prominent yellow-orange background.
Hair Diseases	
Alopecia Areata	Black dots are prominent in SoC trichoscopy and signify disease activity, while yellow dots are more common in light-skinned patients. Both groups display exclamation mark and broken hairs as key trichoscopy signs.
Trichotillomania	Trichitillomania in both groups presents with characteristic broken hairs and black dots under trichoscopy.
Miscellaneous	
Juvenile Xanthogranuloma (JXG)	JXG in both groups exhibits the “setting sun” sign and “clouds” of paler yellow areas on dermoscopy.
Infantile Hemangioma (IH)	Vascular lacunae and coiled vessels are consistent in both populations, though SoC may show a more prominent red–blue background. Homogeneity of color and size variation are shared dermoscopy features.
Port Wine Stains (PWSs)	PWSs in both groups exhibit mixed vascular patterns and white circles, with the latter characteristic appearing especially in facial lesions. SoC dermoscopy images of PWSs can present with a pink hue, especially in children.
Pyogenic Granuloma (PG)	SoC PG images often show more prominent serum crusting. Images of PG on SoC and in mixed-population studies display red homogeneous areas with white rail lines and collarettes, while ulcerations and vascular structures are seen across both populations.

## Data Availability

The data described in this study are available upon request from the corresponding author.
